# A novel association between Bmi-1 protein expression and the SUVmax obtained by ^18^F-FDG PET/CT in patients with gastric adenocarcinoma

**DOI:** 10.1515/biol-2022-0087

**Published:** 2022-12-09

**Authors:** Ying Guo, Li Zhang, Qingjie Ma

**Affiliations:** Department of Nephrology, China-Japan Union Hospital, Jilin University, 126 Xiantai St. Changchun, Jilin 130033, China; Department of Neurology, China-Japan Union Hospital, Jilin University, 126 Xiantai St. Changchun, Jilin 130033, China; Department of Nuclear Medicine, China-Japan Union Hospital, Jilin University, 126 Xiantai St., Changchun, Jilin 130033, China

**Keywords:** gastric cancer, gastric adenocarcinoma, Bmi-1, GLUT1, ^18^F-FDG PET/CT, SUVmax

## Abstract

This study aimed to examine B-cell-specific Moloney murine leukemia virus integration site 1 (Bmi-1) in gastric adenocarcinoma (GAC) and its association with the maximal standard uptake value (SUVmax) of preoperative fluorine-18-fludeoosyglucose positron emission tomography/computed tomography (^18^F-FDG PET/CT). Clinicopathological data were retrospectively collected from 60 primary GAC patients. The Bmi-1 protein expression in GAC and adjacent noncancerous tissues was examined by immunohistochemistry and western blot analysis. Pearson’s correlation analysis was conducted to assess the correlation between Bmi-1 expression and the SUVmax. The Bmi-1 protein levels were significantly greater in GAC versus noncancerous tissues, and higher Bmi-1 was significantly correlated with a lower degree of tumor differentiation, higher tumor stages, more lymph node metastasis, and depth of invasion. The SUVmax value was significantly correlated with the T stage, N stage, and clinical stage, but not with age, gender, tumor size, histological differentiation degree, or Lauren classification. Moreover, a significant positive correlation between Bmi-1 and SUVmax was observed in GAC tissues. In conclusion, our findings demonstrate a novel correlation between Bmi-1 and preoperative SUVmax in GAC patients who did not receive radiotherapy, chemotherapy, or targeted treatment before surgery, and both are positively correlated with unfavorable prognostic factors and a higher grade of malignancy.

## 1 Introduction

Gastric cancer (GC) remains one of the most common malignancies worldwide, and as many as 930,000 cases are diagnosed annually [1]. It has been noted that the incidence rate of GC is particularly high in many countries of East Asia, including China [2]. Although its incidence is high, the 5-year survival rate of GC is relatively low, ranging from 20 to 35% [3,4,5]. Studies also have shown that GC patients with distant metastasis who did not receive surgical treatment have a 5-year survival rate of less than 10% and a median overall survival time as short as 10–14 months [6,7]. The majority of GC patients are diagnosed at late stages, which is considered to contribute largely to the poor prognosis [3,4]. Despite substantial progress in the development of chemotherapy in combination with trastuzumab (IgG1 monoclonal antibody targeting the human epidermal growth factor receptor 2 [HER2]), which has been demonstrated to result in an improved survival rate of patients with positive HER2 expression, the current treatment options for advanced GC patients are limited [8]. Therefore, a better understanding of the molecular mechanisms underlying GC and the development of targeted therapies for GC are urgently needed.

Several previous studies have reported that the B-cell-specific Moloney murine leukemia virus integration site 1 (Bmi-1), known as a transcriptional repressor in the PcG family, is overexpressed in a range of human cancers [9,10,11]. Notably, in GC patients, elevated Bmi-1 expression is closely correlated with unfavorable prognostic factors, including large tumor size, differentiation, invasion, lymph node metastasis, and distant metastasis [12,13,14]. Based on these exciting findings, Bmi-1 has been proposed as a new biomarker for GC and may represent a novel target in the development of targeted therapy for GC.

Fluorine-18-fludeoosyglucose positron emission tomography/computed tomography (^18^F-FDG PET/CT) is an imaging technique used to acquire metabolic and anatomical data that has been widely used in the diagnosis, staging, prognostic prediction, and determination of the treatment response of GC patients [15,16,17,18]. Several recent studies have shown that the uptake of FDG, a characteristic metabolite measured by ^18^F-FDG PET/CT, is significantly correlated with the expression of various tumor biomarkers, including epidermal growth factor receptor, Ki-67, and programmed cell death ligand 1 [19,20,21]. However, until now, the relationship between metabolic characteristics (e.g., maximal standard uptake value [SUVmax]) obtained by ^18^F-FDG PET/CT and Bmi-1 protein expression in GC patients is unknown.

In this study, we attempted to examine the Bmi-1 protein expression in primary gastric adenocarcinoma (GAC) tissues in comparison with noncancerous specimens among patients undergoing surgical resection and to assess its association with the SUVmax obtained by ^18^F-FDG PET/CT in GAC.

We present the following article in accordance with the STROBE checklist.

## 2 Materials and methods

### 2.1 Study subjects

A total of 60 patients with GAC who underwent preoperative ^18^F-FDG PET/CT scanning before surgical treatment at the China-Japan Union Hospital, Jilin University (Changchun, Jilin, China), spanning the period between September 2015 and September 2019, were retrospectively examined in this study. GAC was histopathologically diagnosed and confirmed by pathologists in the Department of Pathology, the China-Japan Union Hospital, Jilin University. None of the study subjects had received radiotherapy, chemotherapy, or targeted treatment before surgery. The SUVmax was obtained from the preoperative ^18^F-FDG PET/CT scan. Relatively normal tissues, at least 3 cm away from the GAC tissues (paracancerous tissues) and collected from the same 60 GAC patients, were used as the control in the present study.


**Informed consent:** Informed consent has been obtained from all individuals included in this study.
**Ethical approval:** The research related to human use has been complied with all the relevant national regulations, institutional policies and in accordance with the tenets of the Helsinki Declaration and has been approved by the Ethics Committee of the China-Japan Union Hospital, Jilin University.

### 2.2 ^18^F-FDG PET/CT imaging and analysis

The procedures of ^18^F-FDG PET/CT imaging are briefly described as follows. After fasting for 4–6 h and having blood glucose levels ≤8 mmol/L, the study patients were intravenously given ^18^F-FDG with a radiochemical purity of ≥95%, and the body weight-adjusted dose was 5.3 MBq/kg. After drinking water (300–500 mL), resting for 50–60 min, and emptying the bladder, PET/CT scans were performed using an uMI50 PET/CT integrated system (Shanghai Lianying Medical Technology Co., Ltd.) with emission scans in the three-dimensional (3-D) mode. For each study patient, a PET scan was performed through the whole body. The following parameters were used for PET scanning: acquisition mode, 3-D scanning; voltage, 120 kV; current, 50 mA; slice thickness, 3 mm; scanning, 5–7 bed positions with 3 min per bed position. A CT scan was subsequently conducted from the base of the skull to the middle segment of the femur. After image acquisition, the PET images were attenuation-corrected by the CT data. The PET images were reconstructed, and the images were integrated using the workstation software (Shanghai Lianying Medical Technology Co., Ltd.). The PET/CT images included the following three types: CT images, PET images, and integrated PET/CT images. All images were analyzed by two independent senior radiologists, who defined the region of interest, also referred to as the volume of interest, and the average SUVmax value of the primary GAC was calculated. A positive status was defined as a high concentration of radioactivity in the primary tumors, i.e., SUVmax ≥2.5.

### 2.3 Immunohistochemical examinations

Immunohistochemistry was performed to examine the protein levels of Bmi-1 and GLUT1 in the 60 surgically resected GAC specimens and the 60 surgically removed paracancerous tissues as relatively normal gastric specimens. Horseradish peroxidase-conjugated goat anti-rabbit IgG (Jackson ImmunoResearch), Bmi-1 primary antibody, and GLUT1 primary antibody (Affinity) were used in the immunohistochemical analysis according to the standard protocol. In brief, 4-mm-thick tissue sections were embedded in paraffin, dewaxed, and rehydrated. The sections were incubated with 3% hydrogen peroxide for 25 min and then with 10% normal goat serum at room temperature. The sections were subsequently reacted with the primary anti-Bmi-1 or GLUT1 antibody at 4°C overnight. The resulting sections were washed three times with phosphate-buffered saline (PBS) (5 min each time) and then incubated with the secondary antibody for 30 min at room temperature. After washing completely with PBS, the sections were incubated with streptavidin peroxidase for 10 min at room temperature (UltraSensitiveTM SP [Rabbit] IHC Kit) and rinsed three times with PBS (3 min each time). The sections were developed using diaminobenzidine. After counterstaining with hematoxylin for 2 min, dehydrating, and clearing with xylene, the sections were mounted and analyzed for Bmi-1 or GLUT1 protein expression in the tissues. The optical density, which reflected the amount of Bmi-1 or GLUT1, was examined using Image-Pro Plus software, and the values in three different fields were obtained from each section to quantify Bmi-1 or GLUT1 protein expression.

### 2.4 Western blot analysis

Western blot analysis was conducted to examine the protein expression of Bmi-1 in 60 primary GAC tissues and 60 matched cancer-free specimens. Briefly, 100 mg of tissue was homogenized in 200 µL of lysate buffer and centrifuged at 4°C for 10 min. The supernatants were used for the bicinchoninic acid assay to quantify the protein concentration. Total protein samples (30 µg) were separated by sodium dodecyl sulfate–polyacrylamide gel electrophoresis and transferred onto nitrocellulose membranes, followed by blocking with 5% nonfat milk. The membranes were then incubated overnight with a primary antibody at 4°C. The dilutions of primary antibodies were as follows: 1:400 for anti-Bmi-1 antibody and 1:1,000 for anti-GAPDH antibody. The membranes were subsequently incubated for 1 h with horseradish peroxidase-conjugated secondary antibody (dilution: 1:5,000) at room temperature. The proteins were visualized using electrochemiluminescence reagents, and the intensity of the Bmi-1 or GAPDH band was analyzed using Gel-Pro Analyzer software. The Bmi-1 protein expression was normalized to that of GAPDH.

### 2.5 Statistical analysis

Statistical analysis was carried out using SPSS19.0 software (IBM) (Chicago, IL, USA). The measurement data were expressed as the mean ± standard deviation (SD). For data with a normal distribution, the *t*-test was conducted for comparison between groups. Analysis of variance (ANOVA) in combination with the *post hoc* Turkey’s honestly significant difference test was used to compare data of multiple groups. The correlation between two continuous variables was examined by performing Pearson’s correlation analysis. *P* < 0.05 indicated a statistically significant difference.

## 3 Results

### 3.1 Bmi-1 protein expression in GAC and noncancerous tissues

**Figure 1:**
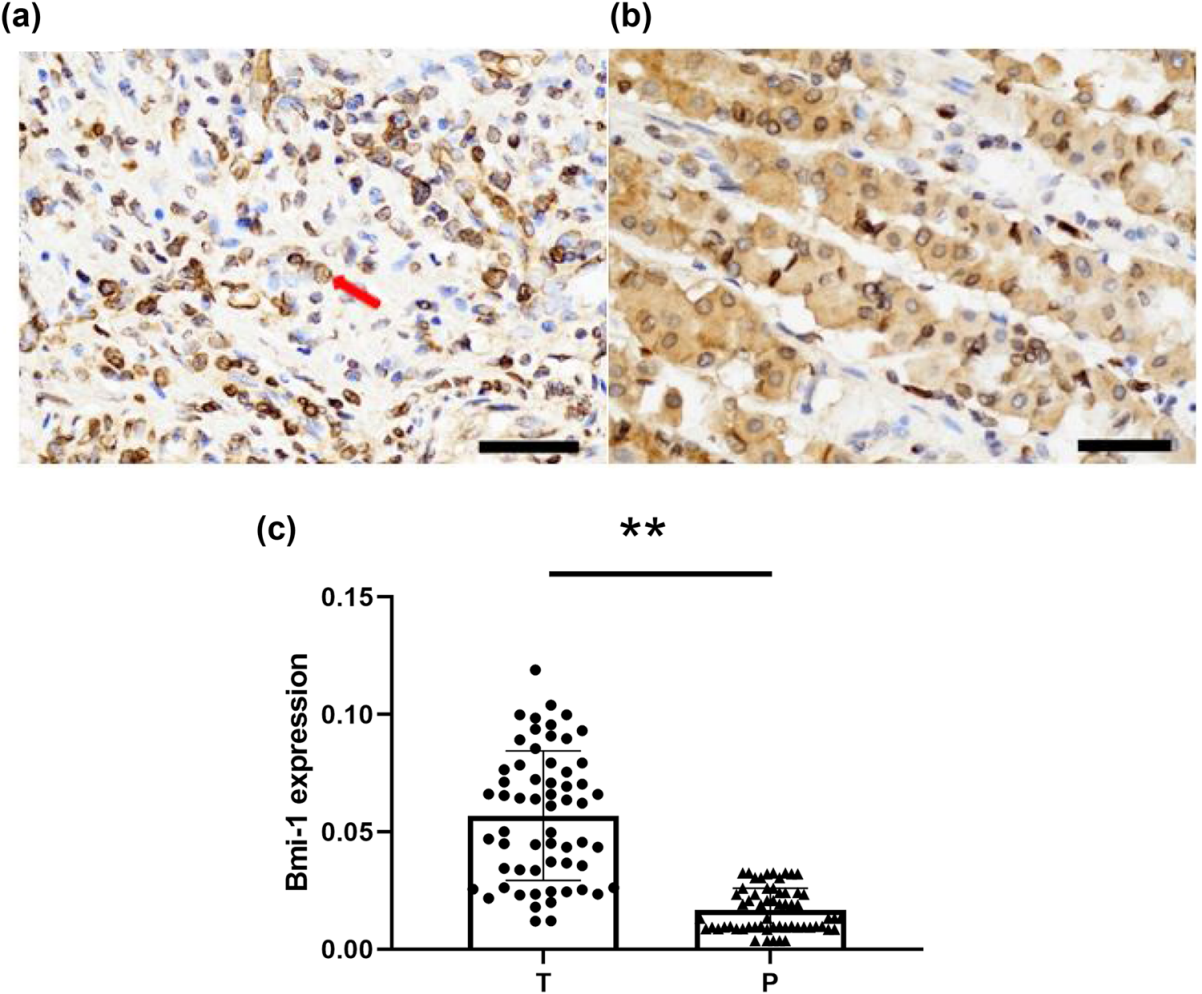
Immunohistochemical analysis of Bmi-1 protein expression in GAC and adjacent noncancerous tissues. Representative immunohistochemical images of Bmi-1 protein expression in (a) GAC and (b) adjacent noncancerous tissues (magnification, 400×). The yellow-brown color represents positive Bmi-1 expression in the nucleus (arrows). (c) Quantitative analysis using Image-Pro Plus software of immunohistochemical images showing Bmi-1 protein expression in the 60 GAC tissues and 60 matched noncancerous specimens. Bmi-1, B-cell-specific Moloney murine leukemia virus integration site 1; T, tumor tissues; P: paracancerous, noncancerous tissues; **P < 0.0001 indicates a significant difference versus noncancerous tissues.

**Figure 2:**
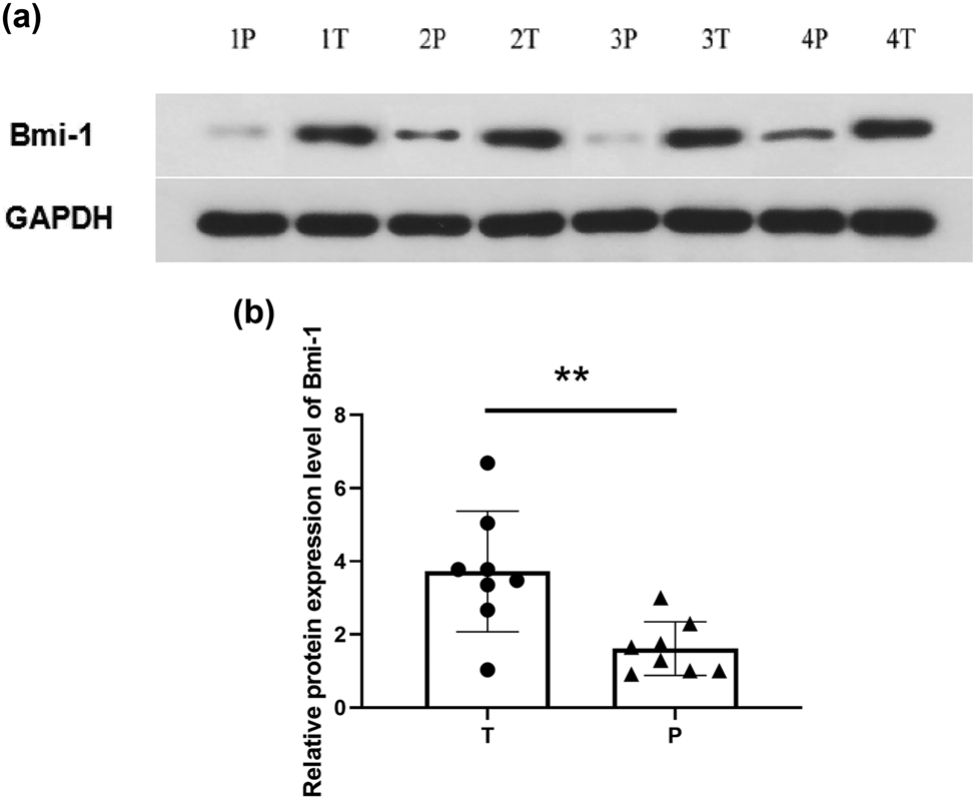
Western blot analysis of Bmi-1 protein expression in GAC and matched noncancerous tissues. (a) Four representative western blot images of Bmi-1 and GAPHD protein expression in four GAC and matched noncancerous specimens; (b) quantification of the Bmi-1 protein levels. The Bmi-1 protein levels were normalized to those of GAPDH. Bmi-1, B-cell-specific Moloney murine leukemia virus integration site 1; GAPDH, glyceraldehyde-3-phosphate dehydrogenase; T, tumor tissues; P: paracancerous, noncancerous tissues; **P < 0.05 indicates a significant difference versus noncancerous tissues.

Immunohistochemistry and western blot analysis were performed to examine the protein expression of Bmi-1 in the GAC and noncancerous tissues. As shown in Figure 1a and b, Bmi-1 was mainly found in the nucleus, and its protein expression was elevated in GAC. Further quantitative analysis of the immunohistochemical images for Bmi-1 protein expression in the 60 GAC and 60 matched noncancerous tissues showed that the mean optical density of Bmi-1 protein was 0.056 (SD, 0.026) in the GAC tissues, which is significantly greater than the mean optical density of 0.017 (SD, 0.009) observed in the noncancerous tissues (*P* < 0.0001) (Figure 1c). Similarly, western blot analysis showed that the expression of Bmi-1 was significantly greater in the GAC tissues in comparison with that in the adjacent noncancerous tissues (*P* = 0.0052) (Figure 2). The results of the western blot analysis were in agreement with those of the immunohistochemical examinations, indicating an abnormal increase in the Bmi-1 protein expression in GAC.

### 3.2 Correlation analysis of Bmi-1 and characteristics of the study subjects

**Figure 3:**
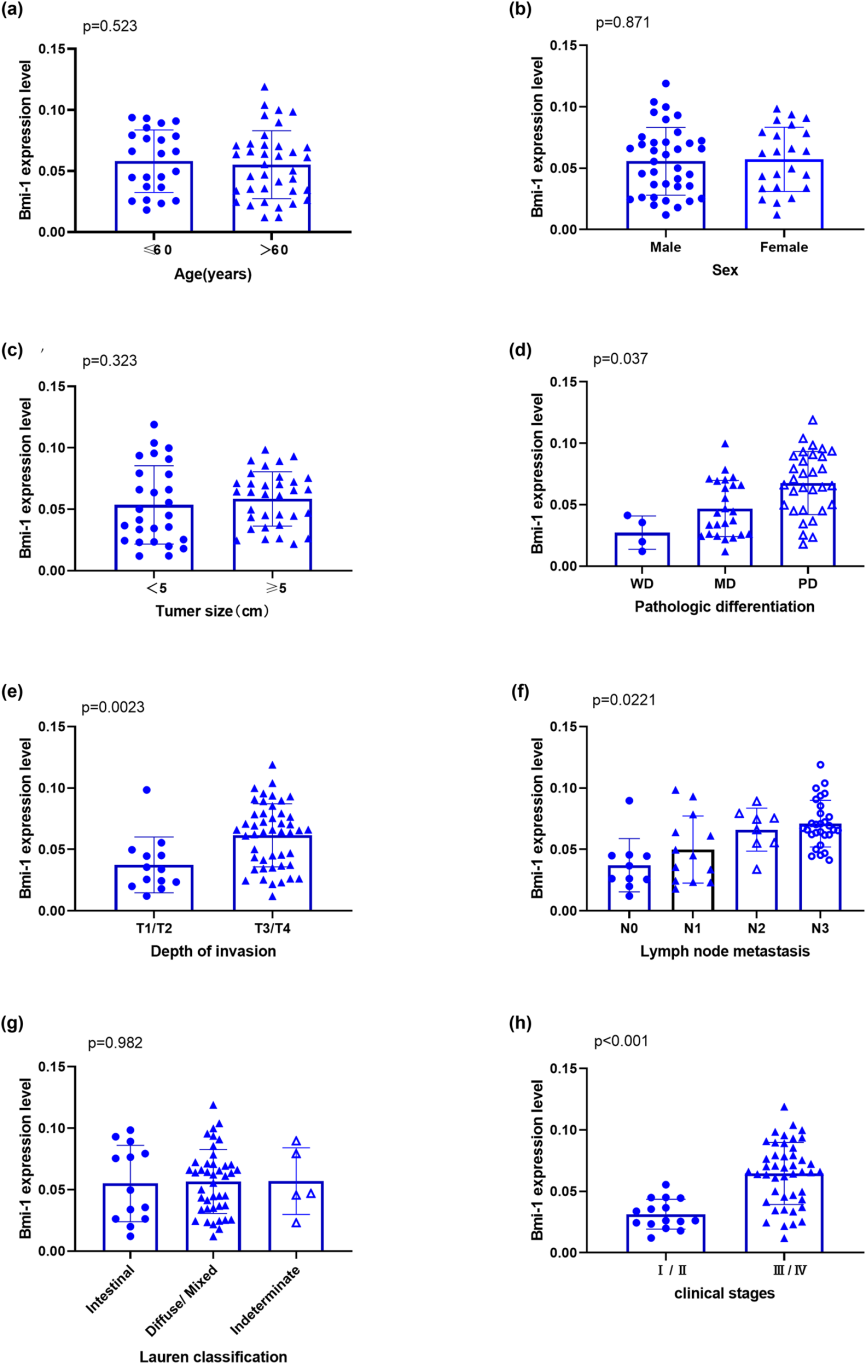
Bmi-1 protein expression in the GAC tissues according to the characteristics of the study subjects. Sixty GAC patients were stratified into different subgroups according to their demographic and clinical characteristics, including (a) age, (b) gender, (c) tumor size, (d) pathological differentiation, (e) depth of invasion, (f) lymph node metastasis, (g) Lauren classification, (h) clinical stage, and the Bmi-1 protein levels were compared between subgroups.

The GAC patients were classified into different subgroups based on their demographic and clinical characteristics, and the Bmi-1 protein expression levels in the GAC tissues of the subgroups were examined by immunohistochemistry (Figure 3). The associations between the Bmi-1 protein expression and various characteristics were assessed. Of note, the levels of Bmi-1 protein were significantly positively correlated with the degree of tumor differentiation (*P* = 0.035), T stage (*P* = 0.0023), N stage (*P* = 0.0221), clinical stage (*P* < 0.001), lymph node metastasis, and depth of invasion, but not with gender (*P* = 0.871), age (*P* = 0.523), Lauren classification (*P* = 0.982), or tumor size (*P* = 0.323) (Table 1). Furthermore, a higher Bmi-1 protein level was positively correlated with lower differentiation, higher invasion, higher lymph node metastasis, and a higher clinical stage.

**Table 1:** Demographic and clinical characteristics of the study subjects as well as their association with Bmi-1 protein expression

Characteristic		*N* (60)	Bmi-1	*P* value
Age (years)	≤60	23	0.058 ± 0.025	0.523*
	>60	37	0.055 ± 0.027	
Gender	Male	37	0.056 ± 0.028	0.871*
	Female	23	0.057 ± 0.026	
Tumor size (cm)	<5	27	0.054 ± 0.032	0.323*
	≥5	33	0.058 ± 0.022	
Degree of differentiation	Well	4	0.027 ± 0.013	0.035^†^
	Moderate	25	0.047 ± 0.023	
	Poor	31	0.068 ± 0.026	
T stage	T1/T2	13	0.037 ± 0.023	0.0023*
	T3/T4	47	0.061 ± 0.026	
N stage	N0	10	0.037 ± 0.022	0.021^†^
	N1	13	0.050 ± 0.027	
	N2	8	0.066 ± 0.017	
	N3	29	0.071 ± 0.019	
Lauren classification	Intestinal	13	0.055 ± 0.031	0.982^†^
	Diffuse/mixed	42	0.057 ± 0.026	
	Unidentified	5	0.057 ± 0.027	
Clinical stage	I/II	15	0.031 ± 0.012	<0.001*
	III/IV	45	0.065 ± 0.025	

Note: *t-test; ^†^one-way ANOVA/Turkey’s honestly significant difference test was used to compare each group.

### 3.3 Correlation analysis of Bmi-1 expression and the SUVmax values of ^18^F-FDG PET/CT imaging

**Figure 4:**
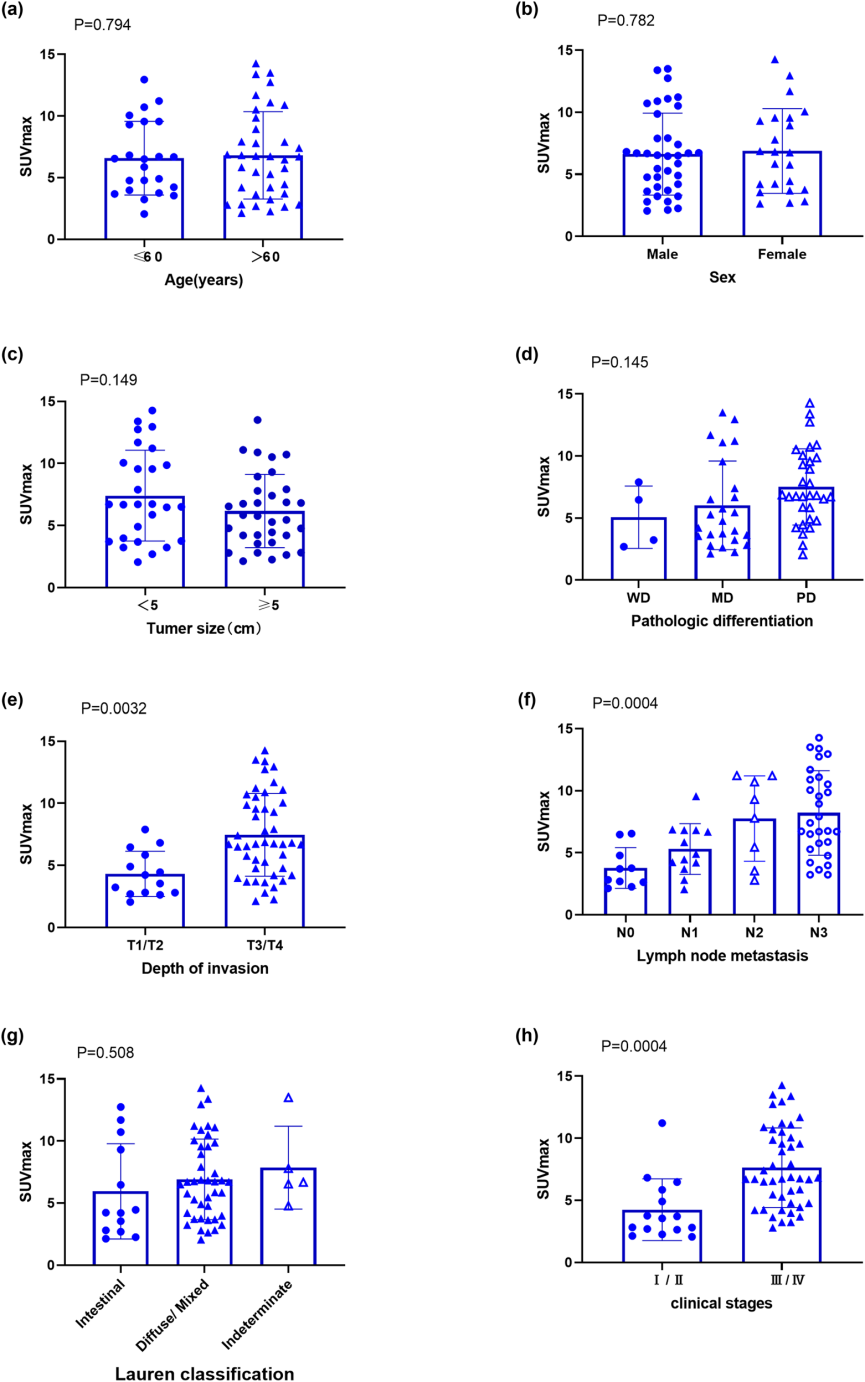
Analysis of the SUVmax values of the GAC tissues according to the characteristics of the study subjects. Sixty GAC patients were stratified into eight subgroups according to their demographic and clinical characteristics, including (a) age, (b) gender, (c) tumor size, (d) pathological differentiation, (e) depth of invasion, (f) lymph node metastasis, (g) Lauren classification, and (h) clinical stages, and the SUVmax values were compared between subgroups. SUVmax, maximum standardized uptake value.

The relationships between the SUVmax values and the clinical characteristics of primary GAC in the study patients were analyzed. Sixty GAC patients underwent ^18^F-FDG PET/CT imaging, and the SUVmax values of the primary focus and various clinicopathological characteristics of GAC were analyzed. As listed in Table 2, the SUVmax values were significantly positively correlated with the clinical stage (*P* = 0.0004), T stage (*P* = 0.0032), and N stage (*P* = 0.0004), but not with gender (*P* = 0.782), age (*P* = 0.794), tumor size (*P* = 0.149), tumor differentiation (*P* = 0.146), or Lauren classification (*P* = 0.982). The SUVmax values were positively correlated with a higher lymph node metastasis, higher tumor invasion, and higher clinical stage (Figure 4).

**Table 2:** Correlation between characteristics of the study subjects and the SUVmax values

Characteristic		*N* (60)	SUVmax	*P* value
Age (years)	≤60	23	6.584 ± 2.986	0.794*
	>60	37	6.816 ± 3.545	
Gender	Male	37	6.632 ± 3.302	0.782*
	Female	23	6.880 ± 3.411	
Tumor size (cm)	<5	27	7.411 ± 3.661	0.149*
	≥5	33	6.167 ± 2.947	
Pathological differentiation	High	4	5.070 ± 2.514	0.146^†^
	Moderate	25	6.022 ± 3.570	
	Low	31	7.510 ± 3.066	
T stage	T1/T2	13	4.314 ± 1.824	0.0032*
	T3/T4	47	7.152 ± 3.291	
N stage	N0	10	3.778 ± 1.645	0.0004^†^
	N1	13	5.301 ± 2.041	
	N2	8	7.758 ± 3.439	
	N3	29	8.213 ± 3.408	
Lauren classification	Intestinal	13	5.942 ± 3.833	0.508^†^
	Diffuse/mixed	42	6.915 ± 3.244	
	Unidentified	5	7.858 ± 3.333	
Clinical stage	I/II	15	4.244 ± 2.487	0.0004*
	III/IV	45	7.629 ± 3.207	

Note: *t-test; ^†^one-way ANOVA/Turkey’s honestly significant difference test was used to compare each group.

**Figure 5:**
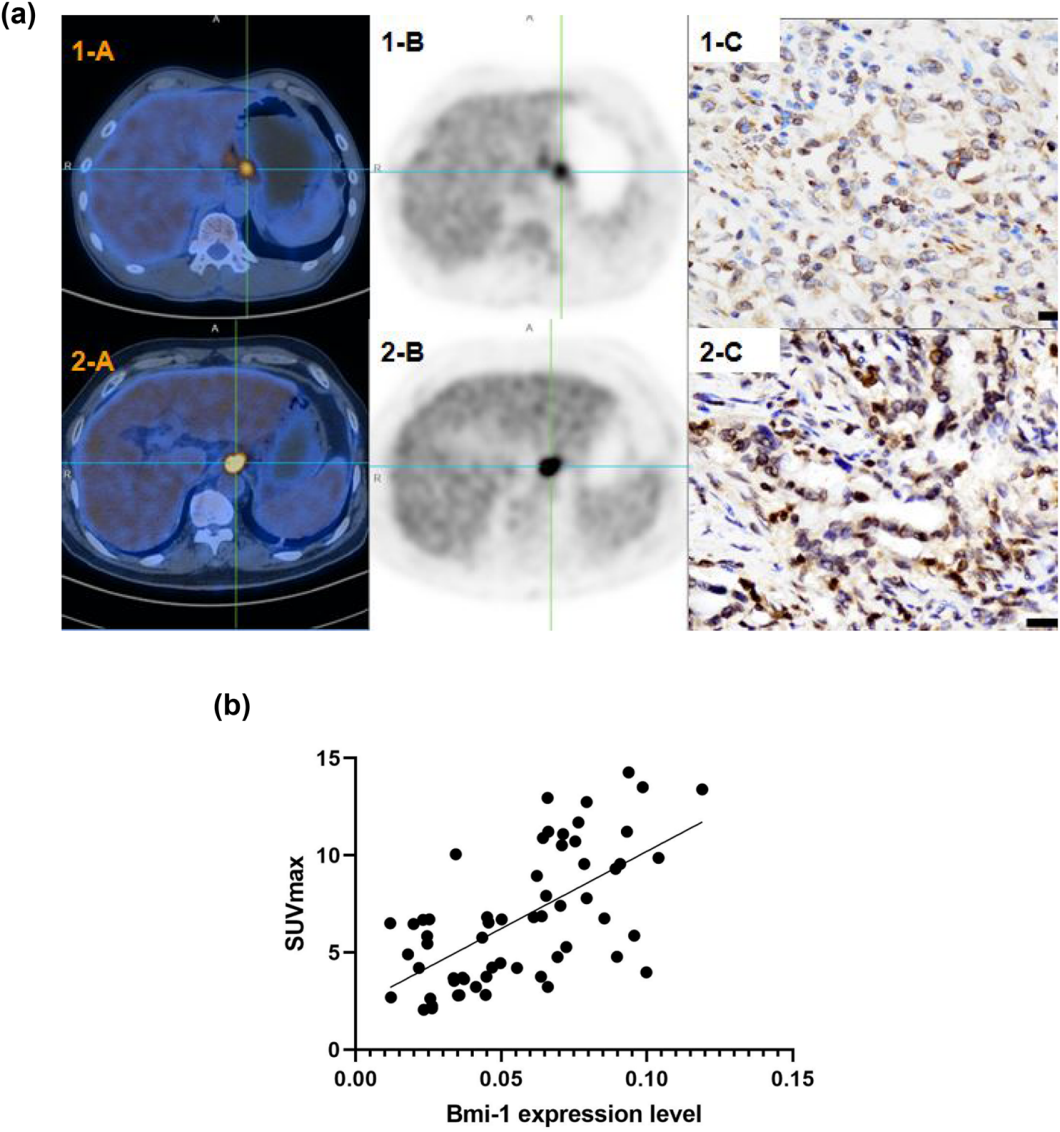
Representative ^18^F-FDG PET/CT images and the correlation between the SUVmax and the Bmi-1 expression in GAC. (a) Top panels from left to right: ^18^F-FDG PET/CT image (1-A), PET/CT image (1-B), and immunohistochemical analysis of Bmi-1 (1-C) in Patient 1 with moderately differentiated GAC and a pathological stage of T2N1MO. Lower panels from left to right: ^18^F-FDG PET/CT image (2-A), PET/CT image (2-B), and immunohistochemical analysis of Bmi-1 (2-C) in Patient 2 with poorly differentiated GAC and a pathological stage of T4N3bMO. In Patient 1, the SUVmax was 5.84, and the optical density of Bmi-1 immunohistochemical staining was 0.423. In Patient 2, the SUVmax was 14.27, and the optical density of Bmi-1 immunohistochemical staining was 0.937; (b) correlation analysis of the SUVmax and the Bmi-1 protein expression in patients with GAC.

Moreover, the correlation between the Bmi-1 protein expression and the SUVmax values was examined. Representative ^18^F-FDG PET/CT and Bmi-1 immunohistochemical staining images of Patient 1 (moderately differentiated GAC) and Patient 2 (poorly differentiated GAC) are illustrated in Figure 5a. The SUVmax was positively associated with the Bmi-1 immunohistochemical staining optical density (SUVmax/Bmi-1, 14.27/0.937 [Patient 2] and 5.84/0.423 [Patient 1]). Based on the ^18^F-FDG PET/CT and Bmi-1 immunohistochemical images of all 60 GAC patients, the correlation between the Bmi-1 immunohistochemical staining optical density and the ^18^F-FDG PET/CT quantitative index SUVmax was assessed, and the results demonstrated that the protein expression of Bmi-1 was significantly positively correlated with the SUVmax values (*r* = 0.629, *P* < 0.0001) (Figure 5b). These findings indicated that the ^18^F-FDG PET/CT imaging of primary GAC in the patients with higher expression levels of Bmi-1 was associated with a higher metabolic rate, while the ^18^F-FDG PET/CT imaging of the patients with lower expression levels of Bmi-1 protein was associated with a lower metabolic rate.

### 3.4 Relationship between BMI-1 expression and GLUT1 in GAC

**Figure 6:**
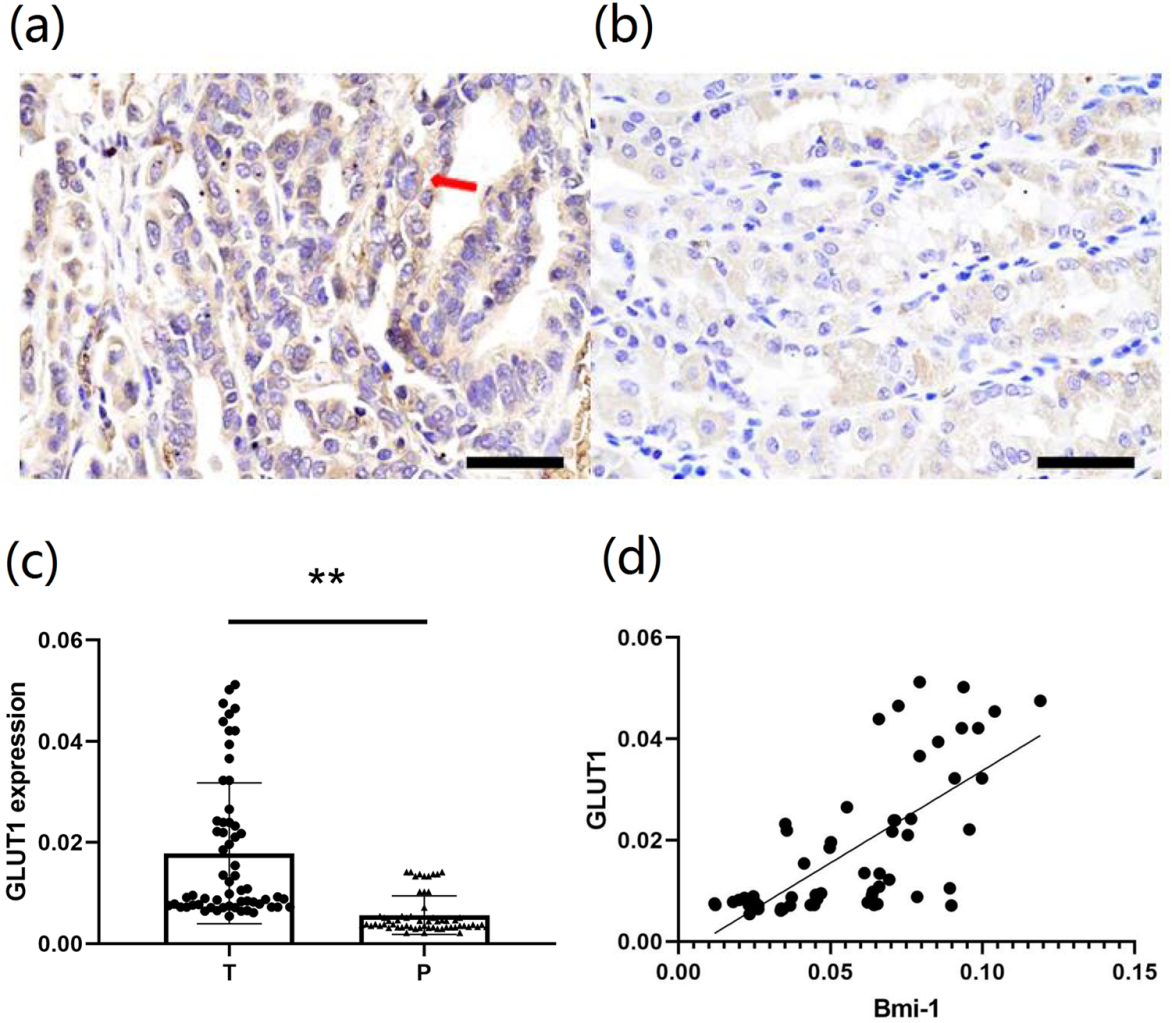
Immunohistochemical analysis of GLUT1 protein expression and its correlation with Bmi-1 protein expression in GAC. Representative immunohistochemical images of GLUT1 protein expression in (a) GAC specimens and (b) noncancerous tissues (magnification, 400×). The brown color denotes positive GLUT1 expression in the cell membrane (arrows). (c) Comparative analysis of GLUT1 protein expression between the 60 GAC tissues versus the 60 noncancerous specimens. (d) Correlation analysis of GLUT1 protein expression with Bmi-1 protein expression in GAC. Bmi-1, B-cell-specific Moloney murine leukemia virus integration site 1; GLUT1, glucose transporter 1; T, tumor tissues; P: paracancerous, noncancerous tissues; **P < 0.05 indicates a significant difference versus noncancerous tissues.

We detected the expression of GLUT1 in 60 GAC tissues by immunohistochemistry. As shown in Figure 6, immunohistochemical analysis of the GLUT1 protein levels showed that the expression level of this protein in GAC was significantly greater than that in the matched, noncancerous tissues (*P* = 0.0003) (Figure 6). Correlation analysis also identified a significant correlation between the GLUT1 and Bmi-1 protein levels (*r* = 0.7032, *P* < 0.0001).

## 4 Discussion

The main novel findings of the present study on the Bmi-1 expression and its association with the SUVmax obtained by ^18^F-FDG PET/CT in patients with GAC are summarized as follows: (1) Bmi-1 protein expression was significantly higher in primary GAC tissues compared to that in noncancerous specimens; (2) there were significant associations between a high Bmi-1 protein expression and unfavorable prognostic characteristics of GAC; (3) Bmi-1 protein expression was positively correlated with the SUVmax obtained by ^18^F-FDG PET/CT in patients with primary GAC; (4) Bmi-1 protein expression was positively correlated with GLUT1 protein expression in patients with primary GAC. Altogether, these results provide strong evidence of an association between Bmi-1 protein expression and the SUVmax obtained by ^18^F-FDG PET/CT in GAC, and both were positively correlated with a higher grade of malignancy or unfavorable prognostic factors in patients with GAC.

In this study, we initially evaluated the levels of Bmi-1 protein in patients diagnosed with primary GAC, the most common gastric malignancy, and its overexpression was evidenced by both western blot analysis and immunohistochemistry. Furthermore, we noted that higher levels of Bmi-1 protein were correlated with a higher grade of GAC as well as unfavorable prognostic factors, including lymph node metastasis (N stage), tumor infiltration (T stage), and low differentiation. These findings are in agreement with previous studies of Bmi-1 in GC [22,23]. Bmi-1 has been shown to promote the proliferation, invasion, and metastasis of GC, exhibiting a positive correlation with the development and progression of GC [22,23]. For example, Wu and colleagues analyzed a total of 352 primary GC specimens and reported an association between Bmi-1 protein expression and the T stage as well as the malignancy grade [22]. In addition, Chen et al. found that a higher Bmi-1 protein expression level was significantly correlated with a lower overall survival rate as well as a poorer clinical outcome for GC patients undergoing postoperative chemotherapy. Of note, our results regarding the association between the Bmi-1 protein expression level and a higher grade of GAC or unfavorable prognostic factors are consistent with these previous studies [22,23].

Recently, hybrid PET/CT has been used as an important imaging modality in clincial practice during the preoperative medical evaluation of cancer patients [24,25,26]. In this study, PET/CT with ^18^F-FDG was performed to determine the SUVmax, which is a marker of tumor glucose metabolism. It merits attention that there was a significant positive correlation between the SUVmax value and Bmi-1 protein expression, suggesting the usefulness of ^18^F-FDG PET/CT in the evaluation of the Bmi-1 protein expression in primary GAC. Given that the Bmi-1 protein expression is associated with the clinicopathological characteristics of GAC, the assessment of its expression may have important clinical significance. Bmi-1 may serve as a potential prognostic factor for GAC or could be a target molecule in the development of targeted therapy for GC. In this study, the SUVmax values of primary GAC showed a correlation with the grade of GAC malignancy, which is also an unfavorable factor for a poor prognosis of GAC, as observed in previous studies [15,16,17,18,27,28].

Until now, the relationship between Bmi-1 protein expression and ^18^F-FDG PET/CT in GC has not been explored. Although the mechanisms underlying the association remain unclear, our results suggest that Bmi-1 could be involved in the glucose metabolism of GAC cells, as ^18^F-FDG PET/CT is an imaging technique based upon glucose metabolism. The intracellular uptake of FDG is mainly related to the expression of GLUT1, which plays a role in mediating glucose transport, and its expression and activity are regulated by oncogenes and growth factors [29,30,31,32]. It has been noted that GLUT1 expression is strongly associated with tumor progression and the survival rate of patients [33,34,35]. In our study, Bmi-1 protein expression was positively correlated with GLUT1 protein expression in patients with primary GAC, suggesting that Bmi-1 could be involved in the regulation of glucose metabolism of tumor cells via GLUT1.

Our study has several limitations that must be addressed. First, the sample size was relatively small, and the clinical outcome data had already been collected in this retrospective study; in addition, the relationship between Bmi-1 protein expression and the overall survival rate was not assessed in the current study. Second, additional bias may have been introduced due to the nature of the retrospective study. As such, it is worth performing a prospective study with a large sample size in the future. Third, although an association between Bmi-1 protein expression and GLUT1 protein expression was identified, the underlying mechanisms remain to be elucidated in the future via in-depth investigations, especially whether and how Bmi-1 exerts its regulatory role on glucose metabolism in GAC.

## 5 Conclusion

Taken together, our findings indicate a novel association between Bmi-1 protein expression and the SUVmax in primary GAC patients who did not receive radiotherapy, chemotherapy, or targeted treatment before surgery, suggesting the usefulness of ^18^F-FDG PET/CT for the evaluation of the Bmi-1 protein expression. Furthermore, both the Bmi-1 protein expression and the SUVmax could be potential prognostic factors for GAC as both are correlated with unfavorable prognostic factors and a higher grade of malignancy. Our results also implicate that Bmi-1 holds promise as a target molecule in the development of targeted therapy for GAC.
